# Xanthene Food Dye, as a Modulator of Alzheimer's Disease Amyloid-beta Peptide Aggregation and the Associated Impaired Neuronal Cell Function

**DOI:** 10.1371/journal.pone.0025752

**Published:** 2011-10-05

**Authors:** H. Edward Wong, Inchan Kwon

**Affiliations:** 1 Department of Chemical Engineering, University of Virginia, Charlottesville, Virginia, United States of America; 2 Institute on Aging, University of Virginia, Charlottesville, Virginia, United States of America; Federal University of Rio de Janeiro, Brazil

## Abstract

**Background:**

Alzheimer's disease (AD) is the most common form of dementia. AD is a degenerative brain disorder that causes problems with memory, thinking and behavior. It has been suggested that aggregation of amyloid-beta peptide (Aβ) is closely linked to the development of AD pathology. In the search for safe, effective modulators, we evaluated the modulating capabilities of erythrosine B (ER), a Food and Drug Administration (FDA)-approved red food dye, on Aβ aggregation and Aβ-associated impaired neuronal cell function.

**Methodology/Principal Findings:**

In order to evaluate the modulating ability of ER on Aβ aggregation, we employed transmission electron microscopy (TEM), thioflavin T (ThT) fluorescence assay, and immunoassays using Aβ-specific antibodies. TEM images and ThT fluorescence of Aβ samples indicate that protofibrils are predominantly generated and persist for at least 3 days. The average length of the ER-induced protofibrils is inversely proportional to the concentration of ER above the stoichiometric concentration of Aβ monomers. Immunoassay results using Aβ-specific antibodies suggest that ER binds to the N-terminus of Aβ and inhibits amyloid fibril formation. In order to evaluate Aβ-associated toxicity we determined the reducing activity of SH-SY5Y neuroblastoma cells treated with Aβ aggregates formed in the absence or in the presence of ER. As the concentration of ER increased above the stoichiometric concentration of Aβ, cellular reducing activity increased and Aβ-associated reducing activity loss was negligible at 500 µM ER.

**Conclusions/Significance:**

Our findings show that ER is a novel modulator of Aβ aggregation and reduces Aβ-associated impaired cell function. Our findings also suggest that xanthene dye can be a new type of small molecule modulator of Aβ aggregation. With demonstrated safety profiles and blood-brain permeability, ER represents a particularly attractive aggregation modulator for amyloidogenic proteins associated with neurodegenerative diseases.

## Introduction

Growing evidence suggests that protein misfolding and aggregation closely correlate to the onset of numerous neurodegenerative diseases, such as Alzheimer's disease (AD), Parkinson’s disease (PD), and Huntington's disease (HD). A common pathological hallmark of these neurodegenerative diseases is the accumulation of insoluble protein aggregates in the brain. Amyloidogenic protein associated with AD, PD, and HD is amyloid-beta peptide (Aβ), α-synuclein, and huntingtin protein, respectively. Although the exact cellular and molecular mechanisms of protein aggregation remains unclear, there is increasing evidence supporting the idea that aggregation of peptides/proteins associated with the neurodegenerative diseases have common cellular and molecular mechanisms [1], [2]. Therefore, it was hypothesized that protein aggregates associated with neurodegenerative disease have common structural features. Glabe et al. discovered that an oligomer-specific antibody raised using amyloid-beta peptide recognizes soluble oligomers of other types of amyloids including α-synuclein, insulin, and polyglutamine [1], demonstrating that soluble oligomers of amyloidogenic proteins share common conformation. Numerous small molecules have been tested for their ability to reduce toxic Aβ aggregates [3], [4], [5], [6], [7], [8], [9], [10], [11]. Recently Wanker et al. reported that (-)-epigallocatechin gallate preferentially binds to unfolded monomeric α-synuclein and Aβ and induces formation of non-toxic oligomers, suggesting that small molecules modulate aggregation of amyloidogenic proteins through a common molecular mechanism [7]. However, the modulation of amyloidogenic protein aggregation by the same small molecule via a common mechanism has not been extensively explored. In order to validate this concept, we chose one α-synuclein aggregation modulator, erythrosine B (ER), considering its demonstrated safety profiles evidenced by FDA approval as a food dye [12], [13]. To our knowledge, Aβ modulating capacities of xanthenes dyes including ER have not been reported. Herein, we evaluate the modulating capacities of ER on Aβ aggregation and Aβ-induced impaired cellular reducing activity in neuronal cells, and investigate whether there are any common features in the interaction mode of erythrosine B with between α-synuclein and Aβ.

**Figure 1:**
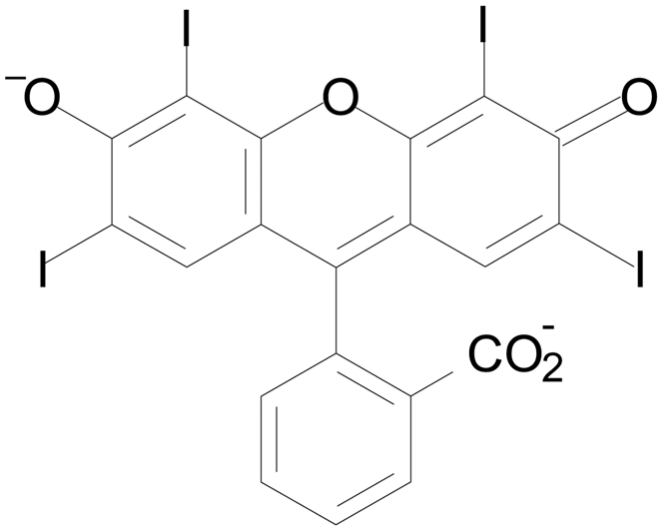
Chemical structure of erythrosine B (ER).

ER is a xanthene dye and is commonly used in coloring candies and cakes (Figure 1). ER is listed in the US as FD&C Red No. 3, in the EU as E127, and also in many other countries as a food coloring dye. It exhibits no observable toxicity up to a daily dose of 149 mg/kg body mass in healthy animals [13]. Likewise, a daily dose of 60 mg/kg does not exhibit any toxicity to humans [14]. ER is highly lipid soluble and so crosses the blood-brain barrier (BBB) [15], [16]. The *in vivo* BBB permeability value of ER is 39 µl/min/g brain, though the condition of the subject can affect plasma protein binding to ER leading to restricted brain uptake [16]. With demonstrated safety profiles and BBB permeability, ER represents a particularly attractive aggregation modulator for amyloidogenic proteins associated with neurodegenerative diseases.

## Materials and Methods

### Aβ Sample Preparation

Aβ40 powder (Anaspec, Inc.) was added to 0.1% trifluoroacetic acid (TFA) to obtain a 1.0 mM stock solution, which was then incubated for one hour without agitation for complete dissolution as described previously [17], [18], [19]. The freshly prepared 1.0 mM stock Aβ solution was diluted with phosphate buffered saline (PBS) solution (10 mM NaH_2_PO_4_ and 150 mM NaCl at pH 7.4) to obtain a 50 µM Aβ solution. 50 µM Aβ samples were then incubated at 37°C for the specified time duration.

### Transmission Electron Microscopy (TEM)

10 µL Aβ sample was spread onto a formvar mesh grid and incubated for one min. The grids were then negatively stained with 2% uranyl acetate for 45 sec., dried and viewed on a Jeol JEM1230 Transmission Electron Microscope (80 kV) located at the Advanced Microscopy Laboratory at the University of Virginia.

### ThT Fluorescence Assay

5 µL of 50 µM Aβ40 sample in the absence or in the presence of ER was diluted in 250 µL of 10 µM ThT in 96-well plates. ThT fluorescence was measured using a Synergy 4 UV-Vis/fluorescence multi-mode microplate reader (Biotek, VT) at an emission wavelength of 485 nm using an excitation wavelength of 450 nm.

### Dot-blotting

2 µL of Aβ samples were spotted onto a nitrocellulose membrane and were dried at room temperature. The nitrocellulose membrane was incubated in 5% skim milk dissolved in 0.1% Tween 20, Tris-buffered saline (TBS-T) solution for one hour. The 5% milk TBS-T solution was removed and the membrane was washed three times (each time for five minutes) with TBS-T solution. The membrane was then incubated with antibody for one hour. Polyclonal A11 anti-oligomer antibody and horseradish peroxidase (HRP)-conjugated anti-rabbit antibody were obtained from Invitrogen (Carlsbad, CA). 4G8 antibody was obtained from Abcam (Cambridge, MA). Monoclonal 6E10 and polyclonal OC antibodies were obtained from Millipore (Billerica, MA). The 4G8, OC, A11 and 6E10 antibodies were diluted in 0.5% milk TBS-T solution according to the manufacturer's protocols. After incubation the membrane was washed three times for 5 minutes using TBS-T solution. In the case of the HRP-conjugated antibody (4G8), membranes were coated with 2 mL of detection agent from the ECL Advance Detection Kit (GE Healthcare, NJ) and the fluorescence was visualized. Otherwise, the membrane was incubated in (1∶5000 dilution in 0.5% milk TBS-T) HRP-conjugated secondary antibody solution for one hour. Then the membrane was washed three times (each time for 5 minutes) with TBS-T solution and the same detection method as previously described was used. The blot images were taken using a BioSpectrum imaging system (UVP, CA).

### MTT Reduction Assay

Viability of Human neuroblastoma SH-SY5Y cells (American Type Culture Collection) was determined by 3-(4,5-Dimethylthiazol-2-yl)-2,5-diphenyltetrazolium bromide (MTT) reduction. 50 mg of MTT (Millipore, MA) was dissolved overnight at 4°C in 10 mL of PBS. The MTT solution was then sterile filtered. SH-SY5Y cells were cultured in a humidified 5% CO_2_/air incubator at 37°C in DMEM/F 12∶1∶1 modified media with 10% fetal bovine serum and 1% penicillin-streptomycin (Thermo Scientific, MA). 20,000 to 25,000 SH-SY5Y cells were seeded into each well of 96-well plates and incubated for 48 hours. Then, the culture medium was replaced with 100 µL of fresh media, and 10 µL of the Aβ sample was added to each well to obtain a final Aβ concentration of 5 µM. Cells were incubated for an additional 48 hours. After replacing the culture medium with a fresh medium, 10 µL of the sterile MTT solution was added, and cells were incubated for 6 hours at 37°C in the dark. After dissolution of the reduced MTT using 200 µL of DMSO, the absorbance was measured at 506 nm using a Synergy 4 UV-Vis/fluorescence multi-mode microplate reader.

## Results and Discussion

### Stabilization of Aβ protofibrils and Inhibition of Aβ fibril formation by ER

In order to monitor morphological changes of Aβ aggregates and formation of amyloid fibrils, we employed TEM and ThT fluorescence assays. TEM has been widely used to obtain morphological information on Aβ aggregates [20], [21], [22], [23]. Aβ intermediates as well as fibrils can be directly visualized with negative-stain TEM. ThT fluoresces at 485 nm when bound to amyloid fibrils [9], [24], [25]. Therefore, ThT fluorescence is used to monitor the progression of amyloid fibril formation. Aβ samples were prepared by incubating 50 µM of Aβ monomer either in the absence (control) or presence of ER from 0 to 3 days at 37°C without shaking as described previously [17], [18], [19].

**Figure 2:**
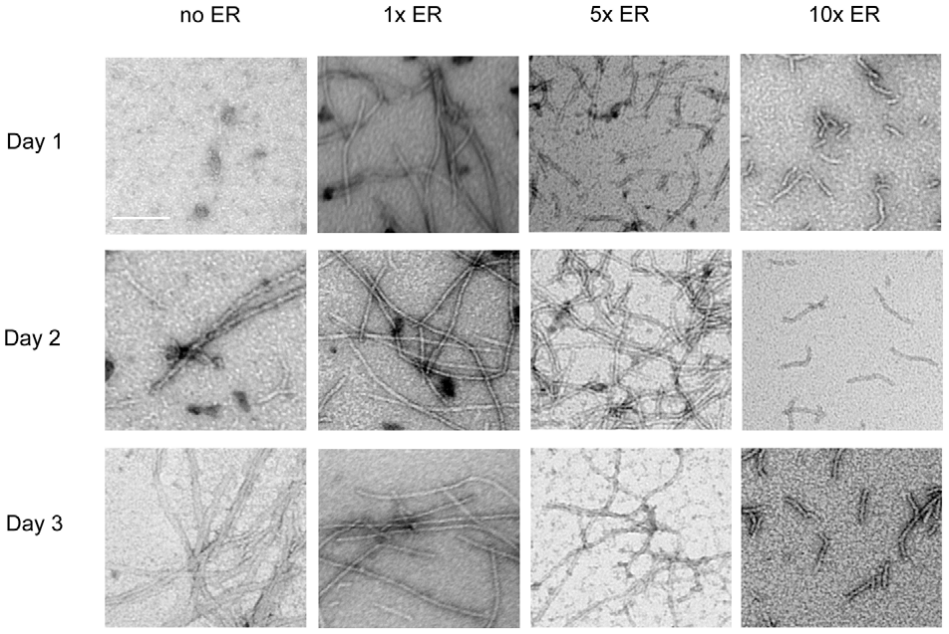
TEM images of Aβ aggregates. Aβ monomers were incubated for one to three days in the absence (no ER) (far-left panels) and the presence of 1x (middle-left panels), 5x (middle-right panels), or 10x ER (far-right panels) and visualized by TEM. Scale bars are 100 nm.

**Figure 3:**
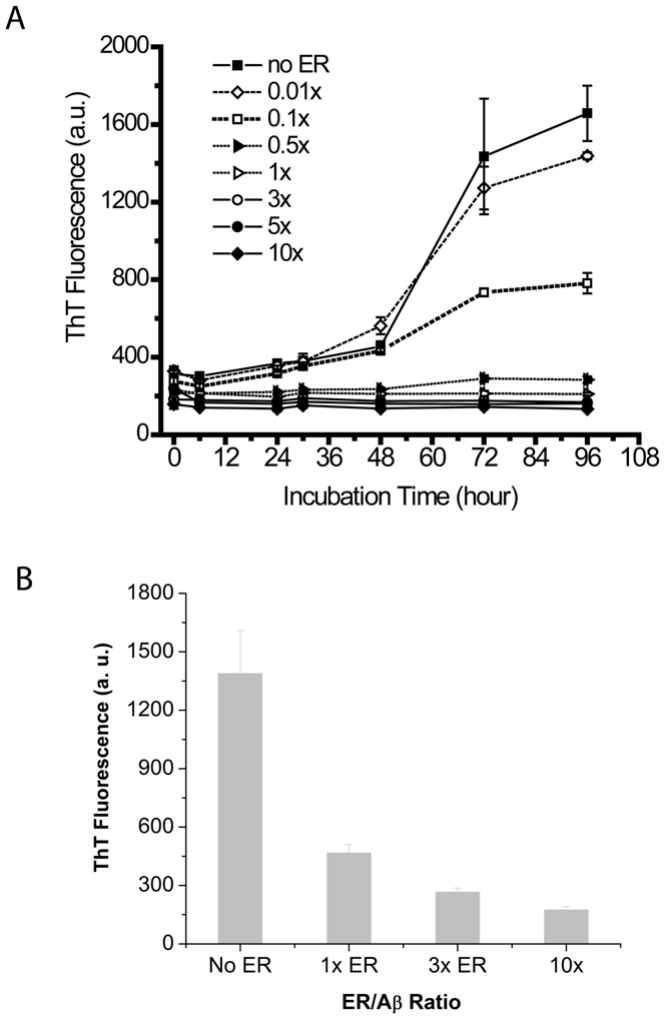
ThT fluorescence of Aβ samples. Aβ monomers were incubated for four days in the absence (no ER) or in the presence of 0.01x, 0.1x, 0.5x, 1x, 3x, 5x, or 10x of ER (A). Preformed amyloid fibrils (72 hrs) were mixed with varying concentrations of ER (1x, 3x, and 10x ER). ThT fluorescence was measured in arbitrary units (a.u.). Values represent means ± standard deviation (n = 3).

Aβ sample TEM images clearly show distinct differences in morphology among Aβ samples incubated in the absence and in the presence of ER (Figure 2). In the absence of ER, oligomers, protofibrils, and amyloid fibril mesh network were sequentially observed throughout the study (Figure 2, far-left panels). The onset of fibril formation at day 2 was confirmed by a dramatic increase in ThT fluorescence from 48 to 72 hr (Figure 3A). At day 1, in the presence of 50 µM of ER (1x ER), protofibrils were the predominant species (Figure 2, top 1x ER panel), whereas oligomers were dominant in the absence of ER (Figure 2, top far-left panel). Furthermore, these protofibrils were observed until day 3 and appeared to have morphological homogeneity characterized by similarity in length and width (Figure 2, 1x ER panels). To evaluate this quantitatively, the length of two hundred Aβ aggregates including protofibrils and fibrils in TEM images at each ER concentration were manually measured using ImageJ (NIH). The length distribution of the two hundred Aβ aggregates at 1x, 5x, or 10x ER concentration is described in Table 1. At day 1, in the presence of 1x ER, the average protofibril length was 690 nm. In the presence of 1x ER, the average length protofibrils did not change substantially on subsequent days (Table 1). At day 1, increasing the ER concentration from 1x to 10x decreased the average length of Aβ aggregates from 690 nm to 55 nm. At days 2 and 3, the average length of protofibrils in the presence of 5x was 758 and 641 nm, respectively, but short protofibrils of length less than 100 nm were predominantly observed in the presence of 10x (Table 1). These findings support the idea that ER promoted the formation of stable intermediate protofibrils.

**Table 1:** Length distribution of Aβ aggregates incubated in the presence of 1x, 5x, and 10x for one, two, and three days.^a^

	ER Conc.	Length of Aβ aggregates (µm)^b^															Average (nm)
		0.1	0.2	0.3	0.4	0.5	0.6	0.7	0.8	0.9	1.0	1.1	1.2	1.3	1.4	>1.4	
Day 1	1x	ND	20	17	27	17	22	11	21	7	13	7	7	9	4	18	690 ± 430
	5x	1	25	68	63	25	15	3	ND	ND	ND	ND	ND	ND	ND	ND	320 ± 110
	10x	187	12	1	ND	ND	ND	ND	ND	ND	ND	ND	ND	ND	ND	ND	55 ± 27
Day 2	1x	ND	9	20	25	24	16	17	18	21	15	9	4	5	2	15	702 ± 424
	5x	ND	ND	1	9	21	28	28	34	28	25	7	3	5	8	3	758 ± 263
	10x	162	37	1	ND	ND	ND	ND	ND	ND	ND	ND	ND	ND	ND	ND	73 ± 37
Day 3	1x	1	16	13	18	15	21	16	20	17	9	9	9	6	5	25	784 ± 485
	5x	ND	3	8	23	25	31	31	33	16	18	4	3	5	ND	ND	642 ± 233
	10x	151	45	4	ND	ND	ND	ND	ND	ND	ND	ND	ND	ND	ND	ND	83 ± 41

a: Two hundred Aβ aggregates observed in negative-stain TEM images at each concentration of ER were analyzed.

b: Each bin has an 100 nm interval in the length of Aβ aggregates. The number indicates the maximum length of Aβ aggregates in each bin.

ND: Not Detected.

Furthermore, ER likely limited the capability to form longer fibrils. Therefore, next we determined whether the inhibition of fibril formation was dependent on ER concentration using ThT fluorescence assay. In the absence of ER, there was a steady increase in the ThT fluorescence of Aβ samples throughout the study (Figure 3A). In particular, a steep increase in the fluorescence was observed from 48 to 72 hr, indicating amyloid fibril formation. However, as the ER concentration varied from 0.01x to 10x, a substantial reduction in the ThT fluorescence was observed (Figure 3A). However, the ThT data of the Aβ aggregates formed with ER should be interpreted with caution due to interference of ER with the ThT fluorescence measurement. In order to investigate the interference of ER with the ThT fluorescence, three different concentrations of ER (1x, 3x, and 10x) were added to the Aβ aggregates incubated without any ER for 3 days displaying a high ThT fluorescence intensity. Addition of 1x, 3x, or 10x ER caused 66%, 81%, and 88% reduction in the Aβ aggregate fluorescence, respectively (Figure 3B), suggesting that ER competitively binds to ThT bindings sites on Aβ fibrils. However, the ThT fluorescence of the Aβ aggregates formed with 1x or 3X ER for 3 days was twice lower than that of the Aβ aggregates at day 3 mixed with 1x or 3x ER respectively, suggesting that the ER-induced Aβ protofibrils weakly bind ThT compared to Aβ fibrils. Glabe et al. also reported that Aβ fibrillar oligomers with stacked β-sheet structure are OC-antibody reactive but weakly bind ThT [26].

In summary, co-incubation of Aβ with ER concentrations of 1x or greater inhibits high-molecular weight Aβ fibril formation and leads to formation of protofibrils and stabilization of the protofibrils at least up to 3 days. At day 1, the average length of Aβ protofibrils is inversely proportional to the concentration of ER at the concentration of 1x ER or greater (Table 1).

### Immuno-reactivity changes of Aβ aggregates by ER

Dot blotting with Aβ-specific antibodies was also used to monitor Aβ aggregate formation. These assays were performed to obtain an integrated picture of Aβ aggregation modulation by ER. With Aβ-specific antibodies, dot blotting has become an effective method for monitoring Aβ aggregate formation [1], [26], [27], [28], [29], [30]. Four Aβ-specific antibodies were utilized for this purpose. 4G8 is an Aβ-sequence-specific monoclonal antibody [31], [32], [33], [34] that binds to amino acids 17 to 24 of Aβ, the hydrophobic patch of Aβ. 6E10 is a monoclonal antibody that recognizes Aβ residues 1-16 [28], [35]. A11 is a polyclonal antibody that reacts with soluble toxic oligomers and protofibrils [7], [28], [30]. OC is a polyclonal antibody that reacts with aggregates with fibrillar structure, including fibrillar oligomers, protofibrils and fibrils [28], [29].

**Figure 4:**
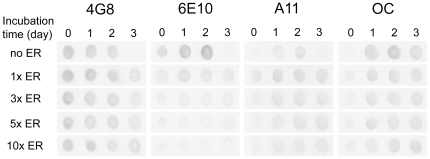
Dot-blotting of Aβ samples using four Aβ-specific antibodies. Aβ monomers were incubated at 37°C in the absence (no ER) or presence of the indicated concentrations of ER (from 1x to 10x) for up to 3 days. Samples were spotted onto a nitrocellulose membrane and immunostained with the 4G8, 6E10, A11, or OC antibody.

**Figure 5:**
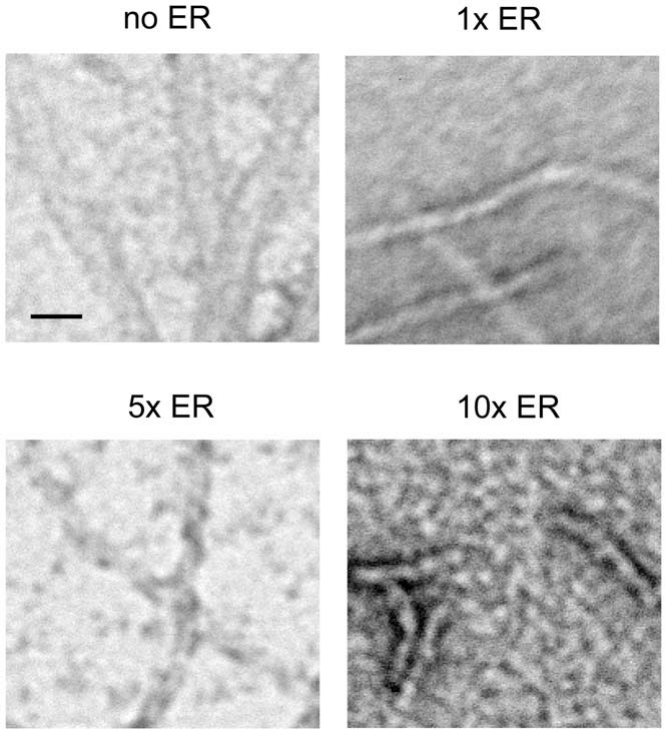
TEM images of Aβ aggregates after three day incubation. Aβ monomers were incubated for three days in the absence (no ER) or in the presence of 1x, 5x, or 10x ER and visualized by TEM. Scale bars are 20 nm.

In the absence of ER, 4G8-reactive species were detected from day 0 to 2, whereas 4G8-reactivity was nearly negligible at day 3. Considering that the 4G8 epitope lies in a hydrophobic patch of the Aβ peptide that is known to be buried during amyloid fibril formation, a dramatic loss of the 4G8 signal was regarded as the onset of extensive fibril-mesh networking [35]. The loss of 4G8 signal corresponding to the formation of fibril-mesh networks was also confirmed by TEM results (Figure 2, far-left panels). In contrast, in the presence of 1x ER or greater, there was prominent 4G8-reactivity even at day 3 indicating that fibril-mesh networks were not readily formed, which is consistent with TEM results. In the absence of ER, significant 6E10 reactivity was observed from day 0 to 2. Under these conditions, the N-terminus of Aβ, the 6E10 epitope, is easily accessible to the 6E10 antibody. At day 3, only very weak 6E10 signal was detected, which is most probably due to Aβ conformation change restricting 6E10 antibody binding similar to 4E8. However, as ER concentration was increased from 1x to 10x, 6E10 reactivity decreased significantly even at day 1 and 2. At the concentration of 10x ER, 6E10 reactivity was significantly weaker than reactivity in the absence of ER (Figure 4). This suggests that ER interaction with Aβ inhibits 6E10 binding. There are two possible mechanisms. First ER competitively binds to the 6E10 epitope. Second, ER alters the conformation of Aβ limiting antibody accessibility to the 6E10 epitope, essentially hiding the N-terminus. According to the structural model of Aβ40 fibril proposed by Grigorieff et. al., two pairs of Aβ protofibrils intertwine adjacently to form a fibril with a 20 nm cross-sectional width [36], which is consistent with the 20 nm Aβ-fibril cross-sectional width observed in Figure 5 (no ER panel). In their model, the N-terminus of each protofibril is laterally exposed and interlocked to form a fibril. Based on this Aβ40 fibril structural model, we speculate that ER binding disrupts the coalescence of two protofibrils leading to inhibition of amyloid fibril formation. This is also consistent with the results that ER-induced oligomers/protofibrils (7 nm cross-sectional width) are stable and do not form fibrils (Figure 5).

In the absence of ER, significant A11-signals were detected at day 1 and 2 but not at day 3 indicating the A11-reactive species were formed until day 2 but converted into amyloid fibrils between day 2 and 3. In contrast, when 1x ER was present, A11-signal was detected even at day 0 and persisted until day 3. In the presence of 3x, 5x, and 10x ER, substantial A11-signal was detected from day 1 to 3, which is consistent with predominant protofibrils but no fibrils were observed in the TEM images. In the absence or the presence of ER, OC-reactive signals remain unchanged (Figure 4) indicating that ER-induced protofibrils still have fibrillar structures. These dot-blotting results support the idea that ER induces formation of both A11- and OC-reactive oligomers but inhibits fibril formation.

### Modulation of aggregation of Aβ and α-synuclein by ER

All of these findings strongly support the idea that ER is an efficient aggregation modulator. ER promotes the formation of Aβ protofibrils and stabilizes them for at least three days, which in turn inhibits fibril formation. Based on the Aβ40 fibril structural model proposed by Grigorieff et. al. and the immunoassay results using the Aβ N-terminus specific antibody, inhibition of Aβ fibril formation is at least partly due to ER binding to the Aβ N-terminus by blocking binding of two protofibrils to form one fibril. As mentioned earlier, it is worthwhile to compare modulation of aggregation of α-synuclein and Aβ by ER. Previous studies on α-synuclein aggregation [12] and our findings indicate that ER promotes protofibril formation of both α-synuclein and Aβ. ER binding sites were found to be predominantly on the hydrophobic region of non-Aβ component of AD amyloid [12]. Therefore, the authors speculate that ER facilitates hydrophoboic interaction of α-synuclein leading to fast protofibril formation. Similarly aromatic rings of ER might play a key role in the promotion of Aβ aggregation at 1x ER concentration. However, although the concentration of ER increased up to 10x, the hydrophobic patch of Aβ (4G8 epitope) was not completely buried, suggesting that the interaction mode of ER with Aβ is different from that with α-synuclein. A bigger difference was found in the effects of xanthene dyes on fibril formation of α-synuclein and Aβ. Although xanthene dyes lead to formation of α-synuclein fibrils, ER clearly inhibits formation of Aβ fibrils. These comparisons suggest that ER promotes protofibril formation of both α-synuclein and Aβ most probably due to the interaction of xanthene aromatic rings with the hydrophobic region of each protein. However, ER is thought to bind to the N-terminus of Aβ leading to the inhibition of Aβ fibril formation, which distinguishes the ER interaction with Aβ from that with α-synuclein.

### Inhibition of Aβ-induced impaired cellular reducing activity by ER

Modulation of Aβ aggregation by ER was clearly demonstrated in the previous sections. However, since Aβ oligomers and protofibrils are normally considered toxic species, we wanted to determine whether ER-induced Aβ protofibrils perturb cellular activities of neuronal cells. In order to determine the detrimental effects of ER-induced Aβ aggregates on cellular functions, we chose the cellular MTT reducing activity of SH-SY5Y neuroblastoma cells. MTT reducing activity has been widely considered as an indication of cell viability [8], [29], [37], [38]. Therefore, the MTT reducing activity loss has often been interpreted as the Aβ-associated cytotoxicity [7], [28], [39], [40], [41], [42], [43]. However, due to a potential issue of the promoted export of the reduced MTT from the cells upon Aβ aggregate treatment leading to reduction of the MTT signal [44], [45], [46], we avoided direct interpretation of the reduced MTT signal as Aβ-associated cytotoxicity but considered the reduced MTT signal as an indication of impaired cellular functions. Cells were adminstered Aβ aggregates preformed in the absence or presence of ER. Preformed Aβ aggregates were prepared by incubating 50 µM Aβ monomers in the absence or presence of various concentrations of ER (1x to 10x) at 37°C for the specified time duration. SH-SY5Y cells were incubated with 5 µM preformed aggregates for 48 hours, and subsequently MTT reducing activity was determined.

**Figure 6:**
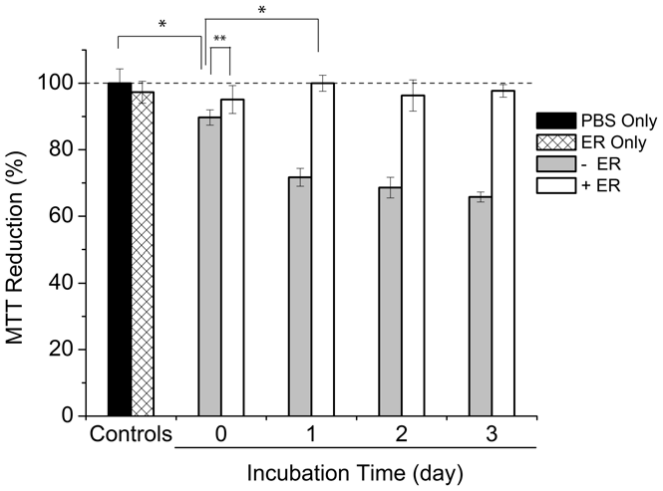
Viability of neuroblastoma SH-SY5Y cells treated with Aβ samples (5 µM) formed in the absence or presence of 10x ER incubated at 37°C for one to three days, measured by MTT reduction. Values represent means ± standard deviation (n≥3). Values are normalized to the viability of cells administered with PBS only. Two-sided Student's t-tests were applied to the data (* P<0.001; ** P<0.005).

**Figure 7:**
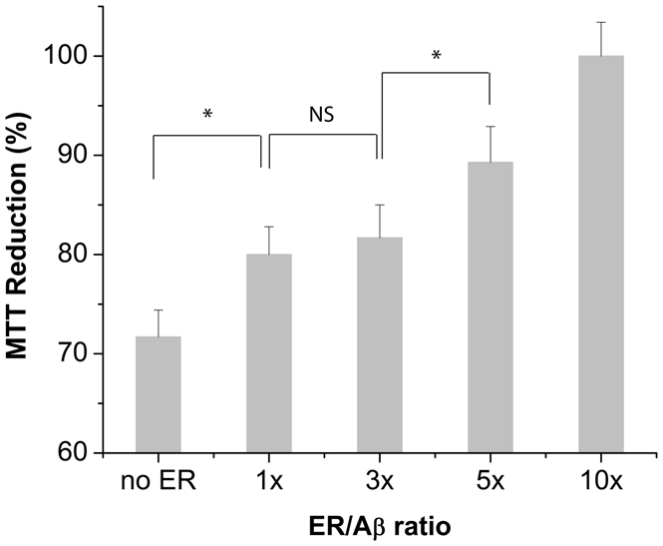
Viability of neuroblastoma SH-SY5Y cells treated with Aβ samples (5 µM) incubated at 37°C for one day in the absence of (no ER) or in the presence of 1x, 3x, 5x, or 10x ER, measured by MTT reduction. Values represent means ± standard deviation (n≥3). Values are normalized to the viability of cells administered with PBS only. Two-sided Student's t-tests were applied to the data (* P<0.001; NS: not significant).

As a control, the MTT reducing activity of cells treated with varying concentrations of ER was measured. Up to 10x ER (500 µM), there was only slight reduction (5%) in the MTT reduction. At day 0, Aβ monomers (5 µM) caused 10% reduction (P<0.001) in the MTT reduction probably due to the toxic Aβ aggregate formation during the Aβ monomer incubation with the cells for 48 hr (Figure 6). However, in the presence of 10x ER, the MTT reducing activity was recovered to 95%. At day 1, preformed Aβ species formed in the absence of ER significantly reduced the MTT reducing activity by 28%. This loss of the MTT reducing activity likely resulted from the formation of both A11- and OC-reactive protofibrils (Figure 4). However, preformed Aβ species prepared in the presence of 10x ER showed an MTT reducing activity significantly higher than those of the Aβ incubated without any ER at days 0 and 1 (P<0.001 and <0.005, respectively) up to the level of a negative control without any Aβ (Figure 6). At day 1, ER reduced the Aβ-induced loss of the MTT reducing activity in a dose-dependent manner (Figure 7). At 1x ER, there was only 8% recovery of the MTT reducing activity (P<0.001). However, the MTT reduction reaches close to 100% in the presence of 10x ER. Similar results were shown with preformed Aβ on subsequent days. The cellulr MTT reducing activity with Aβ (5 µM) incubated for 2 and 3 days decreased to 69% and 66%, respectively. In particular, co-incubation of 10x ER raised the MTT reducing activity to 96 and 98% at day 2 and 3, respectively (Figure 6).

Based on the cellular MTT reduction results, we comprehensively conclude that 10x ER substantially mitigates Aβ-induced impaired MTT reducing activity of neuronal cells. Moreover, these findings strongly support the idea that the majority of protofibrils formed in the presence of 10x ER observed in TEM in fact are not detrimental to neuronal cells, though the protofibrils are both A11- and OC-reactive. These results also suggest that ER mitigates Aβ-associated damage to the cellular function by blocking specific sequence of low molecular weight Aβ species that confers damages to neuronal cells. However, even at 10x ER the protofibrils formed are A11- and OC-reactive, suggesting that ER binding sites on the Aβ do not overlap with the epitope of either A11 or OC antibody.
